# Open bite in adult patients

**DOI:** 10.1590/2177-6709.24.5.069-078.bbo

**Published:** 2019

**Authors:** Carlos Alberto Estevanell Tavares, Susiane Allgayer

**Affiliations:** 1 Associação Brasileira de Odontologia - Seção Rio Grande do Sul, Curso de Especialização em Ortodontia (Porto Alegre/RS, Brazil).; 2 Diplomado(a) pelo Board Brasileiro de Ortodontia e Ortopedia Facial.

**Keywords:** Open bite, Orthodontic anchorage procedures, Malocclusion, Tongue.

## Abstract

Anterior open bite (AOB) is characterized by the lack of overlap or contact between maxillary and mandibular incisors, while the posterior teeth are in occlusion. Correction of this malocclusion is challenging due to difficulties in determining and addressing the etiologic factors, and the high relapse rate. A multidisciplinary approach may be necessary, with participation of Orthodontics, Surgery and Speech Therapy, to achieve adequate esthetic and functional results for long term stability. The present paper discusses the treatment options for AOB, their advantages and implications.

## RESUMO

A mordida aberta anterior (MAA) é caracterizada pela falta de cobertura ou contato entre os incisivos superiores e inferiores, enquanto os dentes posteriores estão em oclusão. A correção dessa má oclusão é considerada desafiadora, devido à dificuldade em se determinar e lidar com os fatores etiológicos e à alta taxa de recidiva. Uma abordagem multidisciplinar pode ser necessária, envolvendo a participação da Ortodontia, Cirurgia e Fonoaudiologia, a fim de que adequados resultados estéticos e funcionais sejam alcançados e se obtenha estabilidade em longo prazo. No presente artigo, o objetivo foi discutir as modalidades de tratamento da MAA, suas vantagens e implicações.

## INTRODUCTION

The etiology of anterior open bite (AOB) is multifactorial, including unfavorable growth patterns, digit-sucking habits, enlarged lymphatic tissue, heredity and oral functional matrices.^1^ ^-^ ^5^ It can impair the speech, swallowing, mastication and esthetics,^1^ thus creating unfavorable conditions for normal social life.^4^ ^,^ ^5^ Depending on the duration, frequency, intensity and age, non-nutritive sucking habits and mouth breathing may cause deformities on the dentofacial complex as a response to the continuous pressure.^2^ ^,^ ^6^

Several treatment options are presented in the literature,^1,7,8^ aiming to inhibit the mechanical factors that maintain the anterior open bite and/or limit the excessive vertical growth of facial skeletal components.^2^ ^,^ ^4^ ^,^ ^9^ ^,^ ^10^ The removal of harmful habits is a complex therapy with psychological, emotional and family involvement. 

Nevertheless, when a patient reaches adulthood without any preventive or interceptive previous treatment, the literature suggests temporary anchorage devices (TADs) or orthognathic surgery associated with orthodontic treatment of severe open bite.^11^

Thus, the present article discusses aspects such as indication and clinical results in the orthodontic-surgical approach for correction of dentofacial deformities caused by AOB. The case report of a patient with Class III malocclusion and severe open bite will illustrate the issue, by demonstrating the favorable esthetic, occlusal and functional results.

## CASE REPORT

The patient, aged 32 years and 5 months, presented the chief complaint of severe open bite and speech problems. The facial photographs showed prominent mandible and increased height of the lower facial third. The intraoral photographs showed Class III relationship, 10-mm severe lateral and anterior open bite, 8-mm overjet and 1-mm upper midline deviation to the left. 

**Figure 1:**
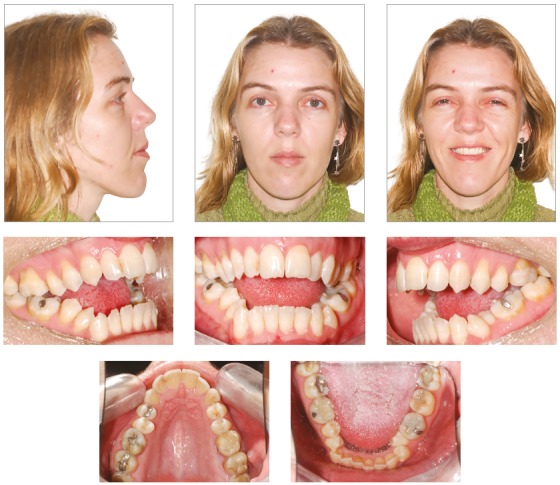
Initial facial and intraoral photographs.

Clinical examination revealed skeletal open bite, infantile swallowing pattern, anterior tongue posture at rest during speech or swallowing, and clicking of the temporomandibular joint. Poor lip sealing with difficult saliva control seriously affected her communication and social interaction, with consequent psychological effects. In maximum intercuspation, occlusal contacts occurred only at the second and third molars and there was full Class III relationship on the left side. There was maxillary atresia with a narrow, V-shaped maxillary arch, with posterior crossbite. The mandibular arch showed negative tooth-size discrepancy of 5 mm (Fig 1). 

Cephalometric analysis revealed Class I sagittal skeletal relationship (ANB = 2^o^). The maxillary incisors were buccally tipped and protruded (1-NA = 11 mm and 1.NA = 25^o^), and the mandibular incisors were retroclined (IMPA = 85^o^). The lower lip was well positioned (LS-LL= 1 mm), and the upper lip was retruded (LS-UL = -3 mm). 

**Figure 2:**
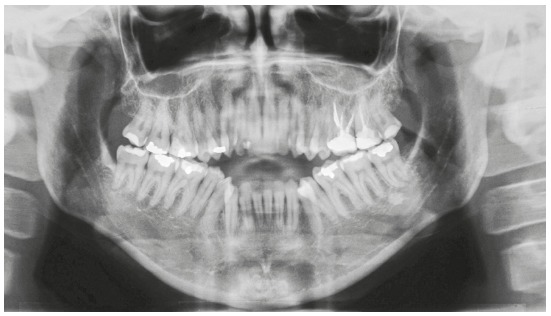
Initial panoramic radiograph.

**Figure 3:**
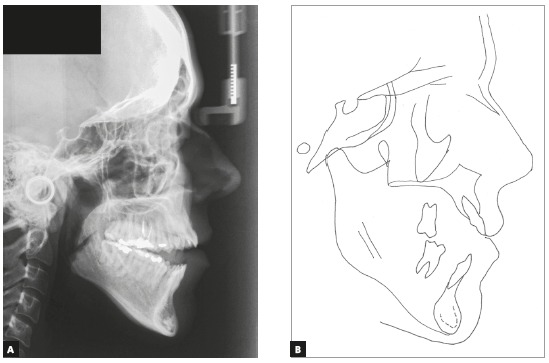
Initial lateral radiograph (A) and cephalometric tracing (B).

In the vertical plane, the skeletal pattern was quite unfavorable (Sn.GoGn = 45^o^, FMA = 35^o^ and Y-axis = 65^o^). Despite the enlarged mandible (Co-Gn = 133 mm) and marked maxillomandibular discrepancy (CoA-CoGn = 47 mm), the McNamara analysis evidenced mandibular retrusion in relation to the cranial base (Pog-Nperp = 10 mm negative) due to an extremely increased lower anterior facial height (LAFH = 96 mm). The panoramic radiograph showed all teeth, including the four third molars (Figs 2 and 3, Table 1). 

**Table 1:** Initial (A) and final (B) cephalometric values.

	Measurements		Normal	A	B	Dif. A/B
Skeletal pattern	SNA	(Steiner)	82°	76°	81°	5
	SNB	(Steiner)	80°	74°	77°	3
	ANB	(Steiner)	2°	2°	4°	2
	Wits	(Jacobson)	♀ 0 ± 2 mm ♂ 1 ± 2 mm	- 9	- 2	7
	Angle of convexity	(Downs)	0°	- 1°	8°	9
	Y-axis	(Downs)	59°	65°	61°	4
	Facial angle	(Downs)	87°	86°	84°	2
	SN-GoGn	(Steiner)	32°	45°	40°	5
	FMA	(Tweed)	25°	39°	33°	6
Dental pattern	IMPA	(Tweed)	90°	85°	93°	8
	1.NA (degrees)	(Steiner)	22°	25°	11°	14
	1-NA (mm)	(Steiner)	4 mm	11mm	2mm	9
	1.NB (degrees)	(Steiner)	25°	28°	30°	2
	1-NB (mm)	(Steiner)	4 mm	8mm	7mm	1
	- Interincisal angle	(Downs)	130°	125°	130°	5
	- Apo	(Steiner)	1mm	3mm	5mm	2
Profile	Upper lip - S-line	(Steiner)	0 mm	- 3mm	0mm	3
	Lower lip - S-line	(Steiner)	0 mm	1mm	1mm	0

### Treatment plan and mechanics applied

The treatment goals were to achieve normal occlusion, correct the vertical and maxillomandibular discrepancy, eliminate crowding, and achieve ideal overjet and overbite, improving function, facial esthetics, and smile characteristics. 

The following treatment alternatives could be considered: a) extracting teeth or distalizing the posterior mandibular teeth using skeletal anchorage^13^ ^-^ ^15^; b) surgically-assisted rapid maxillary expansion (SARME) to improve the narrow maxilla^12^ and conventional surgical orthodontic approach; c) bimaxillary surgery would combine Le Fort I for maxillary repositioning, counterclockwise rotation of the mandible and genioplasty^16^ ^-^ ^19^; or d) early benefit surgery. The latter alternative was refused because it is not fully investigated^20^ ^-^ ^22^ and because relapse after treatment of AOB is quite common.^5,23-25^ Before placement of orthodontic appliance, the patient was referred for evaluation by a speech-language therapist, who detected the need of myofunctional therapy to eliminate the deleterious habits. Orthodontic treatment was divided into two stages: 

» First stage, presurgical, 24 months

**Figure 4:**
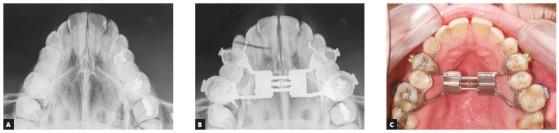
Initial occlusal radiograph (A). Occlusal radiograph (B) and intraoral photograph (C) after surgically-assisted rapid maxillary expansion.

Surgically assisted rapid maxillary expansion (SARME) was performed with a 4-band Hyrax expander activated one turn per day (Fig 4). Simultaneously, during this period, the third molars were extracted, to facilitate distalization of posterior teeth, allowing decompensation of mandibular incisors. 

**Figure 5:**
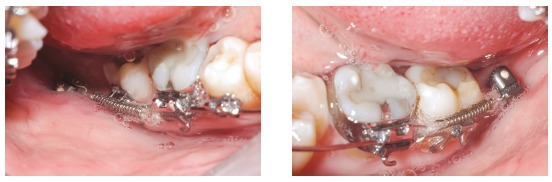
Intermediate intraoral photographs showing TADs during distalization.

Edgewise standard 0.022 x 0.028-in slot brackets were placed in both arches, except for teeth #34, #35 and #44. The typical sequence of archwires, namely 0.0175-in coaxial, followed by stainless steel archwires from 0.014 to 0.020, and 0.019 x 0.025-in (3M Unitek, Monrovia, Calif), was used for alignment and leveling. In the mandibular arch, TADs were installed in the retromolar region, with *Cement-Over O-Ring Abutment* orthodontic configuration (Intra-Lock International MDL Small Diameter Implants, 2.0 x 10 mm, 1.8-mm diameter, Intra-lock^®^ System International Inc., Boca Raton, Florida 33487, USA). Coil springs were connected to the TADs to distalize the mandibular posterior teeth, eliminate crowding and provide alignment and leveling (Fig 5).

Orthognathic surgery was performed including segmented Le Fort I maxillary osteotomy with 7-mm advancement and posterior impaction, as planned in cast surgery and predictive tracing. In the mandible, sagittal osteotomy for counterclockwise rotation was performed, followed by genioplasty for 6-mm setback. The final new jaw positions were stabilized with rigid internal fixation.

» Second stage, postsurgical, 24 months

One month after orthognathic surgery, the patient should wear light posterior vertical elastics full time for three months. The coordination of maxillary and mandibular arches was followed by finishing and detailing of occlusion. The total treatment time was 48 months. During active treatment, the patient underwent monthly speech rehabilitation and myofunctional therapy sessions, to promote correct tongue function.

### Achieved results

**Figure 6:**
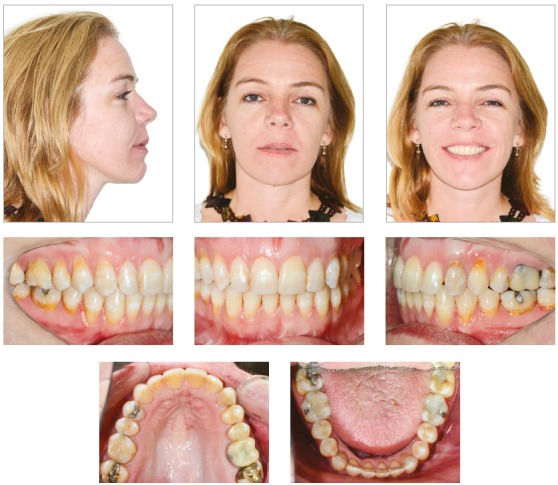
Final facial and intraoral photographs.

The posttreatment photographs confirmed the good esthetic, occlusal and functional results, with Class I molar and canine relationship, ideal overjet and overbite, and adequate incisor display on smile (Fig 6).

**Figure 7:**
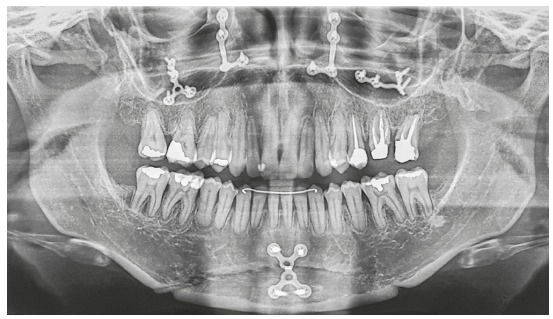
Final panoramic radiograph.

The final panoramic radiograph revealed parallelism and absence of root resorptions (Fig 7).

**Figure 8:**
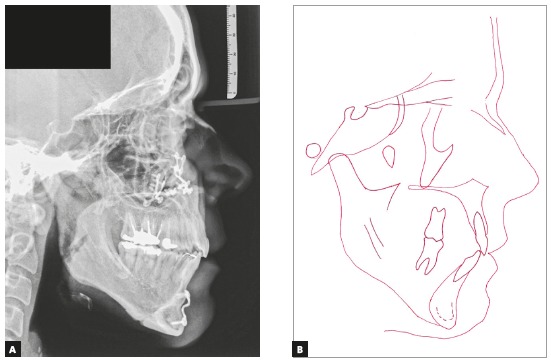
Final lateral radiograph (A) and cephalometric tracing (B).

The most significant cephalometric changes were the 7-mm maxillary advancement and posterior impaction, which led to repositioning of point A and counterclockwise mandibular rotation. As a result, there was improvement in all cephalometric measurements, with harmonious facial profile (Fig 8 and Tab 1).

**Figure 9:**
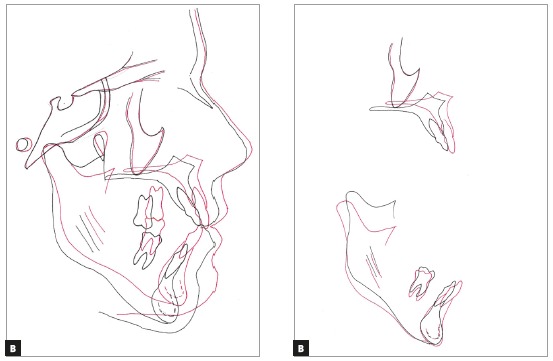
Total (A) and partial (B) superimpositions of initial (black) and final (red) cephalometric tracings.

Cephalometric tracings superimpositions indicated dental and skeletal changes compatible with surgery and the proposed treatment. The results demonstrated distal translation of mandibular molars without extrusion or tipping, thus reflecting distalization of the entire mandibular dentition. The maxillary and mandibular incisors improved their position in the basal bone (Fig 9). 

**Figure 10:**
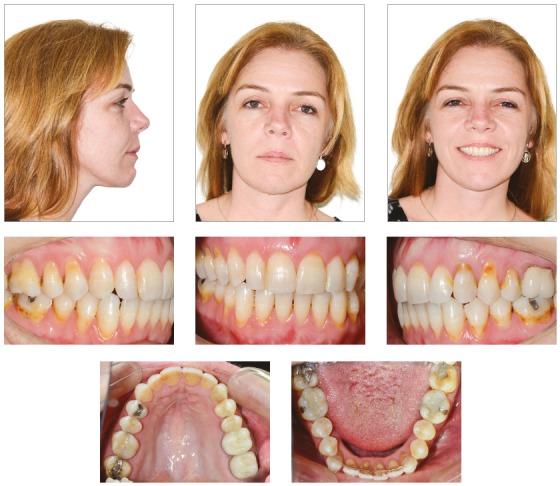
Final facial and intraoral photographs at 2-year follow-up.

Functional occlusion with anterior incisal guidance in excursive movements and canine occlusion on the working side without interference in laterality movement were achieved. Records obtained two years after treatment showed stable results, with slight deviation of the lower midline. Posttreatment stability can also be noticed on the distalized mandibular molar (Fig 10). 

## DISCUSSION

Patients with dentofacial deformities are at disadvantage in society due to low self-esteem, decreased confidence levels, as well as associated physiological problems. This facial and dental deformity impairs the mastication, speech and swallowing, and affects the social behavior of the individual in its multiple aspects,^26^ thus affecting the entire spectrum that constitutes the quality of life.^22,27-30^ Esthetics motivates patients with dentofacial deformity to seek for orthodontic treatment and orthognathic surgery as a treatment option.^31-33^ Knowledge on the patient’s chief complaints and expectations, as well as proper diagnostic exams, are important factors to decide the ideal treatment plan and achieve a satisfactory final result.^34^ ^,^ ^35^

Within this context, Ellis^36^ found that the typical adult with Class III malocclusion clinically appears to have midface deficiency, and the most common combination of variables for thus malocclusion includes retruded maxilla, protruded mandible, protruded maxillary incisors, retruded mandibular incisors and long lower facial height.^36^ In adult patients with skeletal Class III, surgical approach is usually the treatment of choice.^18,29,34,35,37-41^ Thus, a simple surgery may not produce the necessary facial changes for some patients, since some characteristics require maxillary advancement combined with bilateral sagittal osteotomy for occlusal correction and to enhance the patient’s profile.^42-44^ In accordance with these treatment principles and considering that LAFH had the greatest negative impact on facial esthetics in our patient, it was decided to perform a 7-mm maxillary anterior repositioning,^21,42,45^ and posterior impaction, as well as counterclockwise mandibular rotation. The advanced chin resulting from counterclockwise mandibular rotation required a 7-mm genioplasty for setback, thus solving the main problems of this patient, namely excessive LAFH and deficient smile, which were significantly improved. In addition, rotation of the maxillomandibular complex is shown as a valuable alternative to obtain satisfactory esthetic results.^19,42^ The orthognathic surgery straightened the profile, improved the smile, respiratory function, enhanced the self-esteem and thus the quality of life.^22^ ^,^ ^27^ ^-^ ^29^ ^,^ ^45^ ^-^ ^49^

Relapse after treatment is quite common, and the prognosis depends both on the severity and associated etiology.^5,23,24^ Lopez-Gavito et al^25^ reported that more than 30% of patients demonstrated some relapse of anterior open bite after orthodontic treatment. Initially, the tongue posture must be addressed by myofunctional therapy. Also, an appropriate retention protocol is mandatory to avoid the relapse. The present case was managed by myofunctional therapy associated with a maxillary wraparound retainer fabricated with a hole in the palate as a reminder of the normal tongue posture at rest. Due to the complexity of this condition, a multidisciplinary approach involving orthodontist, speech-language therapist, otolaryngologist and surgeon was indicated to achieve the esthetic and functional goals with long-term stability.^5^ ^,^ ^24^

To maximize the stable postoperative occlusion, conventional surgical-orthodontic treatment includes preoperative orthodontics for dental decompensation.^37,39,41,50^ Thus, in this case, appropriate decompensation (Figs 4 and 5) allowed surgical correction without limitation, and orthognathic surgery improved the facial esthetics and provided good jaw relationship for tooth support (Figs 6 to 9). The utilization of TADs mechanics prevented the creation of a negative overjet before surgery and avoided any deterioration in the profile during incisor decompensation. The decompensation can be effectively and efficiently performed with TADs, as shown by the superimposition (Fig 9). Therefore, the need for distal movement of the entire mandibular dentition to solve the mandibular crowding was achieved with TADs without the need of premolar extraction.^50^ ^-^ ^52^

Regarding the multidisciplinary approach, the orthodontist, speech-language therapist and surgeon should communicate on the progress toward surgery throughout the presurgical orthodontic treatment stage. In addition, both orthodontist and surgeon should agree on the presurgical tooth alignment and the desired jaw position after surgery.^20,37^ The professionals should consider the preoperative soft tissue characteristics of patients, to help predict the response of these tissues, mainly concerning the length and fullness of lips, when determining the ideal position of teeth. 

Ultimately, surgery can be used to treat different types of deformities with excellent results. Complications are rare when surgery is done by well-trained, experienced oral and maxillofacial surgeons in well-equipped hospitals. Long-term immobilization provides sufficient time for the muscles to adapt to their new functional length, which is obtained postoperatively^53^. The rigid internal fixation made surgical outcomes more stable and predictable.^20,21,37^ besides allowing earlier postsurgical orthodontic treatment, without the fear of disturbing the new jaw positions.^26^ ^,^ ^54^

Finally, orthodontists should be aware of the orthognathic principles and limits in orthodontic movement and must be experienced and skilled with the skeletal anchorage technique, which is essential to achieve predictable three-dimensional molar movement. The interaction between Orthodontics and Surgery can achieve results that would not be possible if either treatment was applied independently.^44^ ^,^ ^50^ ^,^ ^55^ ^-^ ^56^

## CONCLUSIONS

The standard approach to treat adult patients with dentofacial deformities is the surgical-orthodontic treatment. By careful diagnosis and treatment, the problems diagnosed could be treated effectively and efficiently. The success and stability of treatment of severe AOB depend on an integrated multidisciplinary approach.
